# Cervicovaginal Fungi and Bacteria Associated With Cervical Intraepithelial Neoplasia and High-Risk Human Papillomavirus Infections in a Hispanic Population

**DOI:** 10.3389/fmicb.2018.02533

**Published:** 2018-10-23

**Authors:** Filipa Godoy-Vitorino, Josefina Romaguera, Chunyu Zhao, Daniela Vargas-Robles, Gilmary Ortiz-Morales, Frances Vázquez-Sánchez, Maria Sanchez-Vázquez, Manuel de la Garza-Casillas, Magaly Martinez-Ferrer, James Robert White, Kyle Bittinger, Maria Gloria Dominguez-Bello, Martin J. Blaser

**Affiliations:** ^1^Microbiome Lab, Department of Microbiology and Medical Zoology, School of Medicine, Medical Sciences Campus, University of Puerto Rico, San Juan, PR, United States; ^2^Microbial Ecology and Genomics Laboratory, Department of Natural Sciences, Inter American University of Puerto Rico, San Juan, PR, United States; ^3^Department of Obstetrics and Gynecology, School of Medicine, Medical Sciences Campus, University of Puerto Rico, San Juan, PR, United States; ^4^Division of Gastroenterology, Hepatology, and Nutrition, Children’s Hospital of Philadelphia, Philadelphia, PA, United States; ^5^Department of Biology, University of Puerto Rico, San Juan, PR, Puerto Rico; ^6^Servicio Autónomo Centro Amazónico de Investigación y Control de Enfermedades Tropicales Simón Bolívar, MPPS, Puerto Ayacucho, Venezuela; ^7^Comprehensive Cancer Center, University of Puerto Rico, San Juan, PR, United States; ^8^Department of Pharmaceutical Sciences, Medical Sciences Campus, University of Puerto Rico, San Juan, PR, United States; ^9^Resphera Biosciences, Baltimore, MD, United States; ^10^Department of Biochemistry and Microbiology and of Anthropology, Rutgers University, New Brunswick, NJ, United States; ^11^Department of Medicine and Department of Microbiology, School of Medicine, New York University, New York, NY, United States

**Keywords:** cervicovaginal microbiota, 16S rRNA, ITS2, fungi, cervical cancer

## Abstract

The human cervicovaginal microbiota resides at an interface between the host and the environment and may affect susceptibility to disease. Puerto Rican women have high human papillomavirus (HPV) infection and cervical cancer rates. We hypothesized that the population structure of the cervicovaginal bacterial and fungal biota changed with cervical squamous intraepithelial lesions and HPV infections. DNA was extracted from cervix, introitus, and anal sites of 62 patients attending high-risk San Juan clinics. The 16S rRNA V4 region and ITS-2 fungal regions were amplified and sequenced using Illumina technology. HPV genotyping was determined by reverse hybridization with the HPV SPF10-LiPA25 kit. HPV prevalence was 84% of which ∼44% subjects were infected with high-risk HPV, ∼35% were co-infected with as many as 9 HPV types and ∼5% were infected with exclusively low-risk HPV types. HPV diversity did not change with cervical dysplasia. Cervical bacteria were more diverse in patients with CIN3 pre-cancerous lesions. We found enrichment of *Atopobium vaginae* and *Gardnerella vaginalis* in patients with CIN3 lesions. We found no significant bacterial biomarkers associated with HPV infections. Fungal diversity was significantly higher in cervical samples with high-risk HPV and introitus samples of patients with Atypical Squamous Cells of Undetermined Significance (ASCUS). Fungal biomarker signatures for vagina and cervix include Sporidiobolaceae and *Sacharomyces* for ASCUS, and *Malassezia* for high-risk HPV infections. Our combined data suggests that specific cervicovaginal bacterial and fungal populations are related to the host epithelial microenvironment, and could play roles in cervical dysplasia.

## Introduction

Cervical cancer, one of the most common cancers in women, causes an estimated 11,700 new cases in the United States each year, mostly in black and Hispanic women (Viens et al., 2016). The higher incidence and mortality rates of cervical cancer in Puerto Ricans, US Hispanics, and Blacks compared to Non-Hispanic White women could be due to a higher prevalence of circulating HPV infections coupled with lower screening rates in these groups (Ortiz et al., 2010). Puerto Rican women have a higher age-adjusted incidence for cervical cancer compared to the U.S. mainland (PAN American Health Organization, 2013). Puerto Ricans also have substantial economic and access barriers to treatments, including poor insurance coverage and even fewer credentialed radiotherapy machines island-wide (Kunos et al., 2018). Puerto Rico also has the lowest cervical cancer screening rates in the US, and higher HPV prevalence than on the mainland (34 vs. 27%; Dunne et al., 2007; Ortiz et al., 2013).

Human papillomavirus (HPV) is the etiologic agent for cervical dysplasia and carcinoma (van der Noordaa, 1980; Walboomers et al., 1999) with ∼50 types infecting the epithelium of the genital tract where they can persist asymptomatically or cause neoplasia (Bernard et al., 2010; Doorbar et al., 2012). Transmission of HPV occurs primarily by skin-to-skin contact and likely requires a mild abrasion of the epidermis so the virus can infect the basal cells of the stratified squamous epithelium (Braaten and Laufer, 2008). High-risk HPV (oncogenic) types include HPV 16, 18, 31, 33, 35, 39, 45, 51, 52, and 58 that are associated with cervical, vulvar, vaginal, and anal cancer progression, while low-risk types such as 6, 11, 40, 42, 43, 44, and 54, are associated with genital warts and low grade anogenital tract lesions (Munoz et al., 2003). High-Risk HPV types 16 and 18 together account for ∼70% of cervical cancers, while low-risk types 6 and 11 are responsible for ∼90% of genital warts (Clifford et al., 2003). Persistent infection with high-risk HPV types is a necessary condition for cervical cancer (Nygard et al., 2014). Screening practices include HPV testing in conjunction with cervical cytology (Pap smear) during a pelvic examination with visual inspection of the cervix (colposcopy), especially for women above the age of 30 years (Qaseem et al., 2014). Cervical cytology classification for squamous cell abnormalities includes Atypical Squamous Cells of Undetermined Significance (ASCUS), Negative for squamous intraepithelial lesion (NSIL), Low-Grade, and High-Grade Squamous Intraepithelial Lesion (LGSIL and HGSIL). Cervical histopathology (biopsy) can be manifested as cervical intraepithelial neoplasia (CIN) of grades 1, 2, and 3 for severity (Waxman et al., 2012).

Microbes are predicted to be major actors in malignancies, involving as yet undiscovered mechanisms during infection. As our capacity to estimate the significance of the microbiome in human biology increases, and with results from the Human Microbiome Project (HMP) we now know that 20% of all fatal cancers are microbially induced (Parkin, 2006). The ectocervix is colonized by microbes, whereas the endocervix and the upper genital tract are assumed to be essentially sterile in healthy women (Rampersaud et al., 2012). Changes of the cervicovaginal microbiome, and processes including bacterial vaginosis, cervical inflammation, and increased vaginal pH all affect susceptibility to cervical HPV (Castle et al., 2001). Women of different ethnic groups differ in vaginal microbiome signatures; Hispanic women typically have higher prevalence of communities not dominated by *Lactobacillus* spp. (Ravel et al., 2011).

The majority of the cervicovaginal infections and vaginal discomfort, are due to either bacteria such as *Gardenella vaginalis* or *Atopobium* (Liu M.B. et al., 2013) or by fungi such as *Candida albicans* (Ameen et al., 2017). The cervical epithelium is therefore vulnerable under the influence of infectious microbiome agents, including viruses such as HPV (Widschwendter et al., 2003; Smith et al., 2012; Piyathilake et al., 2016).

Similar to the cervical epithelium, the anal cavity is a physical interface between the host and the environment and mucosal bacteria have been found predict the existence of anal neoplasia, highlighting the relevance of microbiome for the development of new diagnosis and therapeutic targets (Serrano-Villar et al., 2017). With the increasing resolution of modern sequencing methodologies, we now know the microbiome influences cancer susceptibility, in part due to the production of harmful metabolites and their influence on cell function, as a deregulated metabolism and inflammation are hallmarks of cancer (Hanahan and Weinberg, 2011).

Given the importance of oncogenic high-risk HPV persistence in the development of cervical cancer, it is important to perform research on factors that may influence cervical HPV persistence or act as co-factors in CIN. The association of specific microbes (prokaryotic and eukaryotic) with HPV infections and cervical neoplasia remains largely unexplored. The purpose of this study was to examine the association between cervical bacteria and fungi with HPV in varying degrees of CIN in women infected with high-risk (HR) and low-risk (LR) HPVs.

We simultaneously examined the bacterial and fungal biota and genotyped HPV in a sample of Puerto Rican women attending urban San Juan clinics, to determine if their cervicovaginal microbial composition, distribution, and diversity could be related to cervical HPV infection, atypical cell changes, and neoplasia.

## Materials and Methods

### Patient Recruitment

Women coming for gynecology and colposcopy evaluation at the UPR and San Juan City clinics (San Juan Metropolitan area), who did not meet the exclusion criteria, were recruited to participate in this study. Exclusion criteria included: (1) antibiotics taken in the prior 2 months; (2) history of regular urinary incontinence; (3) treatment for or suspicion of prior toxic shock syndrome; (4) candidiasis; (5) active urinary tract infections; (6) active STD; and (7) vaginal irritation at the time of screening. We selected these exclusion criteria based on the indications from the Manual of Procedures of the HMP protocol (McInnes and Cutting, 2010).

The study was approved by the Ethics Committees of the UPR-Medical Sciences Campus IRB (Protocol ref. 1050114/June 2014), San Juan City Hospital and the Inter American University of Puerto Rico IRB (Protocol ref. 1182327-2014). All subjects were informed (both verbally and in writing) of the sampling procedure, risks and benefits of the study, gave written informed consent and signed HIPAA forms, in accordance with the Declaration of Helsinki. All staff involved with the project completed: CITI RCR, Social and Behavioral Research Best Practices for Clinical Research, HIPAA certifications and the NIH training on Protection of Human Subjects. Patients completed a metadata questionnaire with demographic characteristics (age, place of birth, employment, educational attainment), assessment of sexual risk (including age of onset, current sexual partners), health history including antibiotic use, vitamins, and BMI.

### Subject Sampling

As an initial cohort, a total of 62 healthy reproductive-age women (21–50 years old) with the ability to provide informed consent were enrolled in our study. All specimens (vaginal, cervical, and anal) were collected using sterile Catch-All^TM^ Specimen Collection Swabs (Epicentre Biotechnologies, Madison WI, United States), and placed in MoBio bead tubes with buffer (MoBio PowerSoil^TM^ kit, MoBio, Carlsbad, CA, United States) to collect specimens (McInnes and Cutting, 2010). For the introitus (vagina) sampling, labia were spread for visualization, and the specimen was collected by placing one swab in the vagina, posterior to the hymenal ring/tissue, and the swab rotated along the lumen with a circular motion. Swabs were then swirled for ∼20 s in 750 μL of MoBio buffer in the labeled specimen collection tube. For the cervical samples (posterior fornix), a speculum was inserted for access and visualization of the cervix. The sterile swab was placed in the posterior fornix and rotated along the lumen with a circular motion and swabs were immediately placed in the MoBio tubes. Anal specimens were collected by briefly rotating a swab in the mucosa of the anal canal and placement in MoBio collection tubes. All coded vaginal, cervical, and anal swab samples were stored at -80°C and processed for nucleic acid extraction and PCRs at a single laboratory (FGV) to reduce processing variation.

### Genomic DNA Extractions

Genomic DNA extractions were done using the MoBio PowerSoil^TM^ kit, following the manufacturer’s instructions with the following modifications: (1) the powerbead tubes were homogenized horizontally for 2 min at 2,000 rpm, using a PowerLyzer^TM^ 24 Bench Top Bead-Based Homogenizer (MoBio), (2) we combined 100 μl of solution C2 and 100 uL of solution C3 and vortexed for 5 s for cell lysis and, (3) the elution solution was 100 ul sterile PCR water, that was warmed to 65°C, and to increase DNA yield, allowed to remain on the filter for 5 min before the final centrifugation step. The final amount of gDNA ranged from 5 to 40 ng/ul.

### HPV Detection and Genotyping

We used a highly sensitive short-polymerase chain reaction-fragment assay (Labo Biomedical Products, Rijswijk, Netherlands, licensed Innogenetics technology). This assay has been used for epidemiological and vaccination studies, due to high analytical sensitivity (Kleter et al., 1999; Hinten et al., 2017). Briefly, the assay uses SPF10 primers to amplify a 65-bp fragment of the L1 open reading frame of HPV genotypes, followed by a Reverse-Hybridization step. The 65-bp PCR fragment assay amplifies the following common mucosal HPV genotypes: 6, 11, 16, 18, 31, 33, 34, 35, 39, 40, 42, 43, 44, 45, 51, 52, 53, 54, 56, 58, 59, 66, 68/73, 70, and 74. In the second step, the amplified fragments underwent a line probe assay by reverse-hybridization, as described, to determine the specific HPV type compared to the kit-provided controls (Kleter et al., 1999).

### PCR Amplification and Sequencing of Bacterial 16S rRNA and Fungal ITS Genes

For the bacterial communities, we amplified the V4 hypervariable region of the 16S ribosomal RNA marker gene (∼291 bp) using the universal bacterial primers: 515F (5′-GTGCCAGCMGCCGCGGTAA-3′) and 806R (5′-GGACTACHVGGGTWTCTAAT-3′) as described in the Earth Microbiome Project^1^ (Caporaso et al., 2012) using the following amplification conditions: 1 cycle of 94°C for 3 min, and 35 cycles of 94°C for 45 s and 50°C for 60 s and 72°C for 90 s and a final extension of 72°C for 10 min. To ascertain fungal community diversity, we targeted the ITS-2 region using primers ITS9-FW (5′-GAACGCAGCRAAIIGYGA-3′) and ITS4-RV (5′-TCCTCC GCTTATTGATATGC-3′), with amplicon sizes ranging from 240 to 460 bp (de Souza et al., 2016).

16S amplicons were sequenced with a MiSeq Reagent kit 2 bp × 250 bp (Illumina) at the Sequencing & Genotyping Facility of the University of Puerto Rico. The ITS-2 genes were sequenced with a Nextera XT Index Kit (Illumina) at the Louisiana State University (LSU) School of Medicine Genomics Core. We used both positive and negative controls for the library prep processing of 16S and ITS runs. 16S positive controls were mixed bacterial genomic DNA from Microbial Mock Community B (HM-276D, BEI Resources) and the negative controls were from amplicon PCR and index PCRs without DNA templates. All the 16S and ITS raw reads were deposited in the NCBI Sequence Read Archive (SRA) under BioProject accession PRJNA429969.

### Analyses of Sequences

Negative controls from the sequencing run were used to characterize contamination in downstream steps and monitor the quality of the sequencing runs as advised (Kim et al., 2017).

The 16S rRNA gene reads were checked for quality using FastQC (Andrews, 2010) which revealed that only forward reads were useful for downstream analyses. Sequences were de-multiplexed and processed using QIIME (Caporaso et al., 2010), with a requirement of minimum Phred score of 30 for inclusion in the analysis. Chimera filtering was performed with the usearch61 hierarchical clustering method (Edgar et al., 2011). Sequences were clustered into operational taxonomic units (OTUs) using *uclust* (Edgar, 2010) with 97% identity threshold.

Taxonomic assignments were determined using Resphera Insight (Baltimore, MD, United States) (Daquigan et al., 2016, 2017; Guerrero-Preston et al., 2016; Ottesen et al., 2016; Drewes et al., 2017; Grim et al., 2017). This tool relies on a manually curated 16S rRNA database and a hybrid global-local alignment strategy to assign sequences to a high-resolution taxonomic lineage. The approach attempts to achieve species-level resolution, but when the statistical model indicates uncertainty the tool minimizes false positives by providing “ambiguous assignments,” i.e., a taxonomy that is a list of species names reflecting the uncertainty. For example, if a 16S fragment is ambiguous between *Lactobacillus gasseri* and *Lactobacillus hominis*, it is assigned as *“Lactobacillus_gasseri:Lactobacillus_hominis.”* For our analyses, we only retained in the OTU Table single species-level assignments and used the genus level assignments for those sequences not classified at a single species level. We only included samples with more than 1,000 reads for downstream analyses, that were computed in R Development Core Team (2008). Package *qiimer* (Bittinger, 2015) was used to open QIIME output files in R, compute statistics, and create plots from the data. Reads matching chloroplast, mitochondria, archaea, and unassigned sequences were removed. We included only samples that had overall more than 1,000 reads. Biopsy metadata category “Not Recorded” were not used for biopsy analyses, but were used in analyses of all other categories.

Fungal sequence analyses were done using the forward reads (R1) due to their higher accuracy. Primer-dimer reads were removed, as well as the sequences at the beginning of the R1 reads, using in-house scripts. Sequence reads were quality controlled using the QIIME 1.9.0 workflow (Caporaso et al., 2010). OTUs were selected by clustering reads at 95% sequence similarity. All taxonomy assignments were manually curated to check for chimeric/unassigned sequence removal using BLASTn against NCBI’s nt reference (Altschul et al., 1990). Consensus-based taxonomic assignments from the previous BLAST results with the OTU sequence counts for each sample were aggregated at the genus level with BROCC (Dollive et al., 2012)^2^.

In previous studies, a significant problem in interpreting the significance of the fungal microbiota has been the relatively modest numbers of organisms in typical samples and the high representation of contaminating lineages in blank controls, reflecting admixture of fungal DNA from environmental sources during sample acquisition or DNA contamination in reagents (Bittinger et al., 2014). For improved accuracy, we used the post-PCR PicoGreen concentration (prior to amplicon pooling) to annotate the sequencing proportions, to generate an estimation of the OTU abundance considering the ITS abundance in the original DNA specimen.

The corrected abundance of each OTU was calculated by multiplying the post-PCR ITS DNA concentration (PicoGreen) provided by the sequencing facility, with the relative abundance of that OTU in the sample (Bittinger et al., 2014). Only genera present in at least 60% of the samples, with an absolute PicoGreen corrected abundance ≥ 1.0, were used in the analyses. Only samples with at least 1,000 ITS sequence reads were included in the analyses. Downstream data analyses including statistical testing and plots were performed in the R package (R Development Core Team, 2008).

### Taxonomy and Alpha-Diversity

Taxonomic barplots and boxplots showing alpha diversity were built using R’s *ggplot2* package (Wickham, 2009). Alpha diversity measures included richness (number of OTUs) and Shannon index (Shannon and Weaver, 1963). Heatmaps were generated using the *pheatmap* library (Kolde, 2015). Supplementary ubiquity plots were built using the rarefied OTU tables, considering only OTUs that were shared by >40% of the samples. OTU Ubiquity dot plots were built relating OTU relative abundances with OTU proportions among samples (ubiquity). The 16S rRNA and ITS OTU Tables and the metadata file were modified into ubiquity matrices using a Phyloseq-based R code. The OTU table and mapping file were “melted” into four columns: sample ID, metadata variable, taxonomy, and number of reads using the R package *reshape2* (Wickham, 2007). The relative abundance of the OTUs was calculated using the number of sequences in each sample divided by the sum of sequences across samples. The proportion of samples (ubiquity) was calculated using the number of samples that had a given OTU (binary data) divided by the total number of OTUs across all samples. The dot plot was built using R package *ggplot2* (Wickham, 2009) using the facet approach to partition the plot into multiple panels according to the metadata variables.

### Beta Diversity

The abundance of the taxa was analyzed at the community level by computing the pairwise Bray–Curtis distances between samples using the *vegdist* function in the R package *vegan* (Oksanen et al., 2008). The global differences in both bacterial and fungal community composition were visualized with principal coordinate analysis (PCoA).

### Co-occurrence Using the Dice Index

Only the samples with >1,000 reads of both 16S and ITS datasets were retained for the co-occurrence analysis between bacteria and fungi. For the 16S data, OTU sequence counts for each sample were aggregated at species and genus levels. Despite data analyses were done separately for the 16S and ITS datasets, for purposes of showing the variation of bacteria and fungi, we gathered the two BIOM tables (genus level) binding the bacterial genus matrix with the fungal matrix, column-wise, that is in the combined matrix, each sample corresponds to one row, and each genus (either bacteria or fungal) corresponds to one column. On this new matrix the dice index was calculated using the *dist.binary* function from the *ade4* R package (Dray et al., 2017), and end result variable “dist.mat” (matrix). Only the samples with sufficient reads of both 16S and ITS reads were retained for the co-occurrence analysis between bacteria and fungi. For the 16S data, OTU sequence counts for each sample were aggregated at species and genus levels. Taxa candidates were selected if they were present in >50% of the sample groups, and had at least 1% proportion in at least one sample. The Dice index was used to determine the co-occurrence of taxa across sample groups, for any metadata category (Dice, 1945). This metric indicates the degree of association between any two groups. A coefficient close to 1.0 indicates that species being compared are associated more frequently than would be expected by chance alone, and close to 0 indicates that the groups do not co-occur. This was calculated using the *dist.binary* function from the *ade4* R package (Dray et al., 2017), and end result variable “dist.mat” (matrix). The heat map plotting of the distance matrix was done with the *plot_heatmap* function from the *pheatmap* library (Kolde, 2015).

### Statistical Tests

Measures of beta diversity were assessed for differences between sample groups using the PERMANOVA test (Anderson, 2001). The *P*-value in a PERMANOVA test is determined through permutations, and the test statistic is calculated directly from the distance matrix. Analyses of Variance tests, implemented in the *aov* function in R Development Core Team (2008), were used to find significant differences related to a given category. Taxa candidate selection were done if they were present in >50% of the sample groups, and had at least 1% proportion in at least one sample. Biomarker signatures were proposed only if *P*-values were significant after FDR correction (*P* < 0.05). Biomarker signatures were proposed only if *P*-values were significant after FDR correction (*P* < 0.05). The abundance of OTUs was tested to identify signatures significantly associated with the metadata categories (biomarkers). Here, non-parametric Wilcoxon rank sum tests were used, which have a lower false positive rate, are more robust to outliers, and do not require a normal distribution assumption (Fay and Proschan, 2010).

### HPV Diversity and Prevalence

Human papillomavirus diversity was measured using the Hill’s family of diversity numbers (Chao and Jost, 2012; Colwell et al., 2012; Chao et al., 2014) of order 0, 1 and 2, for observed richness, exponential of Shannon entropy index, and inverse of Simpson concentration index following Hsieh et al. (2016) approach with *iNEXT* package in *R 3.3.2* version (R Development Core Team, 2016). The Chao2 metric was used for extrapolated lines -for up to double of the smallest sample size- to estimate the diversity in the Citology, Biopsy, BMI, and age categories. A bootstrap method based on 50 replications was used to build CI_95%_. Non-overlapping values of the CI_95%_ indicated significant differences among groups. HPV prevalence and CI_95%_ plots were generated using *ggplot2* (Wickham, 2009). Comparisons among groups were performed using Fisher’s exact test. Heat maps of HPV type prevalence were built using Excel’s conditional formatting option per different metadata categories, showing cell intensity as proportional to its value.

## Results

### HPV Prevalence and Types

From the 62 HPV genotyped patients, 52 (84.87%) were HPV-positive and 10 (16.13%) were HPV-negative. Of the HPV-positive patients, 27 (51.9%), were infected with, exclusively high-risk HPV types, whereas 3 (5.76%), were infected with exclusively low-risk HPV types. Finally, a total of 22 (42.3%) patients were co-infected by both HPV types (Supplementary Table 1). HPV-type prevalence did not vary significantly between cervical lesions and age (*P* > 0.05, Fisher’s Exact Test) (Supplementary Figure 1).

**FIGURE 1:**
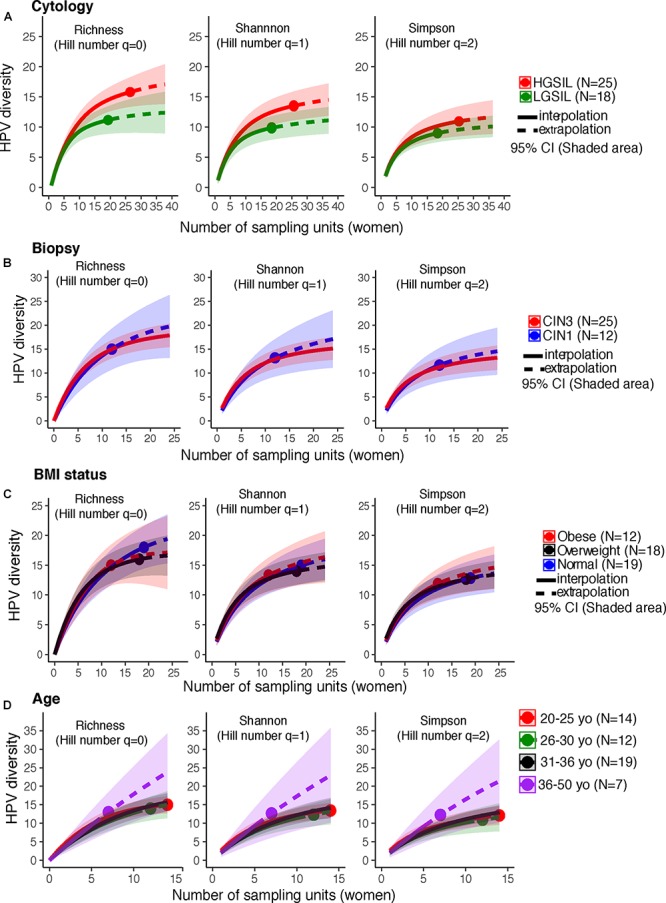
HPV diversity (Shannon index) in cervical samples of HPV+ women according to **(A)** Cytology, **(B)** Biopsy, **(C)** BMI status, and **(D)** age. These analyses were based on a sample size of 52 HPV+ women. There were no significant differences in HPV diversity, according to cytology, biopsy, BMI status, or subject age. The solid line corresponds to an interpolation of the HPV diversity in the 52 women sampled (reached coverage), while the dashed line is an extrapolation to the double of the actual sample size. Comparisons were performed at the end of the extrapolation curves. The shaded areas represent the CI_95%_ estimated from the bootstrap method based on 50 replications.

HPV-16, followed by HPV-18 and 31, were more prevalent in HGSIL (Supplementary Figure 2A). Underweight women had a higher relative abundance of HPV-56 and overweight women had higher HPV-16 (Supplementary Figure 2B). Women with no cervical intraepithelial lesions showed similar HPV-16 prevalence as in CIN3, and as much HPV-51 as CIN1 (Supplementary Figure 2C). HPV-16 was more prevalent in younger women (<30 years old) (Supplementary Figure 2D). There are no significant differences of HPV genotype abundances for any category (*P* > 0.05, Fisher’s Exact Test). We joined the group of women who exclusively had low-risk HPV types (*n* = 3), together with the HPV-negative women (*n* = 10), to form a low-risk HPV group (*n* = 13), comparing them with women who had high-risk types (*n* = 45) (Supplementary Table 2). Co-infections with both high-risk and low-risk ranged from 2 to 9 simultaneous HPV types (Supplementary Table 2). There were no differences in HPV alpha diversity by cervical cytology, biopsy, BMI, or age (*P* > 0.05, CI_95%_, Figures 1A–D).

### Number of Reads and OTUs and Global Patterns of Bacteria and Fungi

We analyzed the microbiota in 58 of the 62 patients, excluding samples with <1,000 reads (Supplementary Tables 3, 4). We recovered a total of 8.4 M high quality reads of bacterial 16S rRNA sequences, and 4.4 M for the fungal ITS region. The 1.8 M vaginal bacterial reads were binned into 3,810 OTUs (965 in high-risk HPV samples and 2,845 in the low-risk samples). In cervical samples, we analyzed a total of 1.9 M bacterial reads, which were binned into 3,080 high-risk associated OTUs and 843 low-risk associated OTUs (Supplementary Table 3). We recovered a total of 1.4 M fungal reads from vaginal samples, which were clustered into 160 high-risk HPV-associated OTUs and 257 low-risk-associated OTUs. The 1.3 M fungal reads in the cervical samples were binned into 412 OTUs (156 in high-risk samples and 256 in low-risk samples) (Supplementary Table 4).

A portion of the samples exhibited a low number of sequence read counts in bacterial 16S sequencing, fungal ITS sequencing and when considering both datasets simultaneously. Prior to analysis of diversity and microbial composition, we eliminated samples having fewer than 1,000 sequence reads simultaneously both the 16S and ITS datasets. For this summary histogram showing the same patients in both datasets, we retained seven subjects in the low risk group (out of the initial 13) and 31 subjects remained in the high risk group (which initially were 45).

**FIGURE 2:**
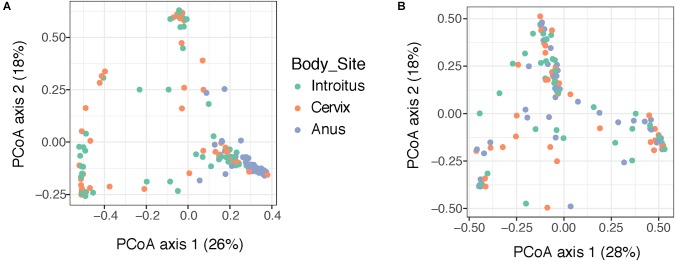
Beta diversity analyses of the bacterial **(A)** and fungal **(B)** communities in 170 patient samples, according to body site. Principal coordinate analyses (PCoA) was built using Bray–Curtis distances. Statistical tests for differences in group centroid position reveal significant differences in bacterial communities across the three body site comparisons (*P* = 0.001), but no significant differences were found comparing fungi (*P* > 0.05) (see Supplementary Table 5).

**FIGURE 3:**
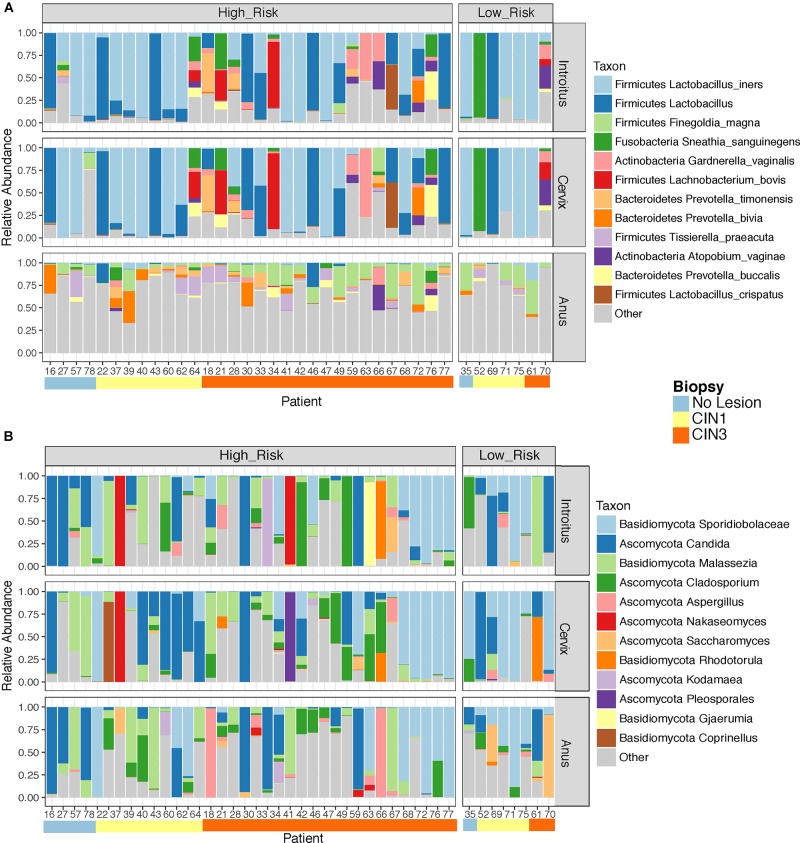
Global taxonomic pattern of bacterial **(A)** and Fungal **(B)** diversity for the dominant taxa in the three body sites examined. We have only shown here patient samples with >1000 reads in both the 16S and ITS datasets simultaneously to maintain the same patients in both analyses.

Beta diversity analyses of all samples according to body site showed significant differences in bacterial community structure (Figure 2A), but no significant differences in the fungal community structure were found with PERMANOVA (Figure 2B and Supplementary Table 5). The global patterns of bacterial populations among body sites showed the expected marked differences between cervicovaginal and anal communities, with the former being dominated by Lactobacilli, and sporadically *Sneathia* and *Gardnerella.* Anal samples were dominated by highly diverse taxa including *Finegoldia, Peptinophilus, Prevotella bivia* and *Prevotella timonensis*, among others (Figure 3A). The global pattern of fungi, contrary to the bacterial site differences, showed very similar fungal patterns among the three body sites, with *Sacharomyces* and *Aspergillus* being more abundant in the anal samples. *Nakaseomyces, Gjaerumia*, and *Pleosporales* were only detected in the cervicovaginal samples but at very low abundance in just few samples (Figure 3B). As anal communities were not impacted by cervical HPV-status or by cervical lesion status (Supplementary Figure 3), and as we had no HPV genotyped in the anal epithelium, henceforth, we focused only on the cervicovaginal samples. *Lactobacillus iners* was the most common high-abundance bacterial taxon, present in more than 83% of the samples, regardless of HPV risk and lesions.

### Cervicovaginal Bacterial Diversity Changes With Dysplasia Severity and Not With HPV Risk

**FIGURE 4:**
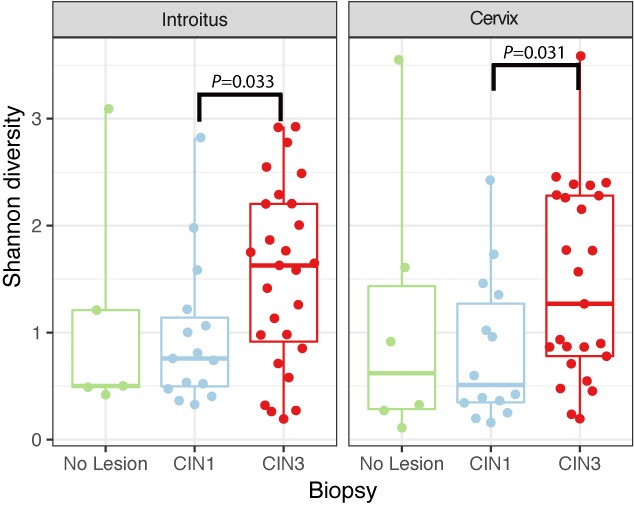
Shannon diversity of bacterial communities in introitus and cervical samples according to degrees of dysplasia. Shannon bacterial diversity was significantly higher in introitus and cervix of CIN 3 compared to CIN 1 patients (*P* = 0.033 and *P* = 0.031, respectively, see Supplementary Table 5).

**FIGURE 5:**
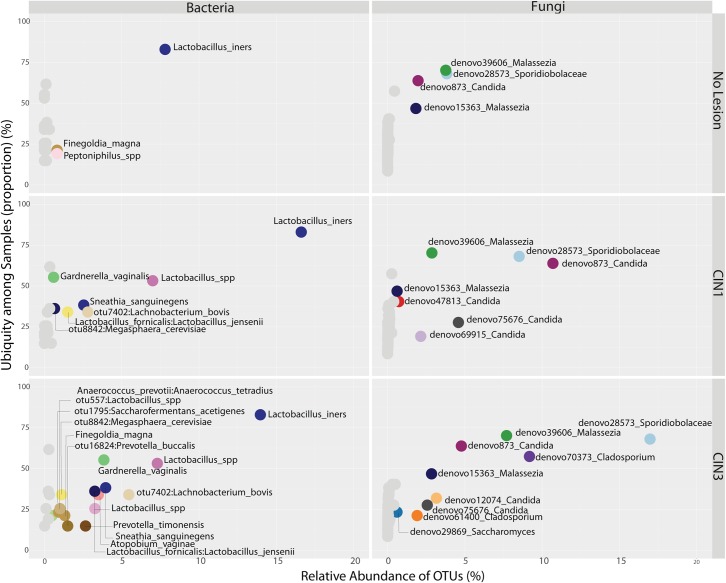
Ubiquity dot plot representing bacterial and fungal OTUs in the cervix, distributed according to their relative abundance and ubiquity, for the different biopsy categories. The analyses were based on the transformation of the OTU table with the *melt* function into single columns for relative abundance, ubiquity and domain using package *reshape2*. The dot plot was built using the *ggplot2* package using the facet approach to partition the plot into multiple panels according to the metadata variables.

Richness was also found to be significantly higher in the cervix in CIN3 lesions compared to CIN1 (Supplementary Figure 5 and Table 5, *P* = 0.03095). The structure of the bacterial community did not change significantly according to either HPV or cervical lesion (Supplementary Figure 5 and Table 5). We found no significant differences in the bacterial diversity, nor did we find specific populations that changed with HPV risk. The Shannon diversity of bacteria was significantly higher in CIN3 compared to CIN1 lesions, both in the vaginal and cervical communities (Figure 4 and Supplementary Table 5, *P* = 0.033 and 0.031 respectively). Although we found no bacterial biomarkers, we found an enrichment in the cervix of *Lactobacillus kitasatonis* and *Tissierella praeacuta* in high-risk HPV samples and *Prevotella timonensis* in CIN3 lesions compared to CIN 1 lesions (Supplementary Figure 6 and Table 5). Other low-dominant taxa such as certain *Gardnerella vaginalis* OTUs, *L. crispatus*, *Prevotella amnii*, and *Parvimonas micra* had consistently higher abundances in CIN3 samples in the cervicovaginal sites and some also reflect that increase in the anal samples (Supplementary Figure 7). The dominant *L. iners*, had relative abundances up to 16% (Figure 5). There were also changes in the relative dominance of several bacterial taxa in the introitus, according to BMI. *L. iners* abundance was decreased in underweight, overweight and obese patients, compared to those with normal weight (Supplementary Figure 8).

### Fungal Cervicovaginal Biota Change According to HPV Risk and Dysplasia Severity

**FIGURE 6:**
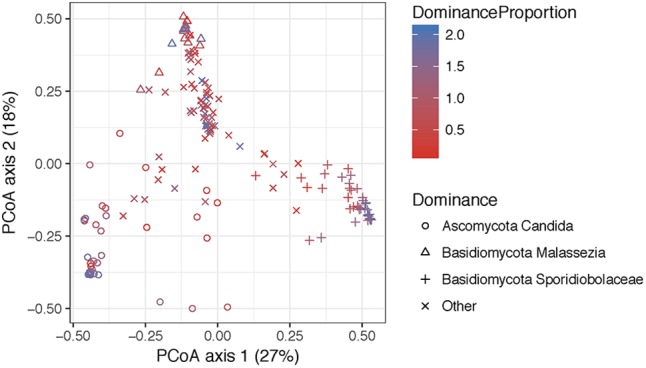
Principal coordinate analyses visualization of pairwise Bray–Curtis distance matrixes of the introitus samples according to most dominant fungal taxa. Points are colored with respect to dominance. Statistical tests for variation in group centroid position reveal significant differences in all three dominant taxa *Candida*, *Malassezia*, and Sporidiolobaceae (*P* = 0.001) (see Supplementary Table 5).

**FIGURE 7:**
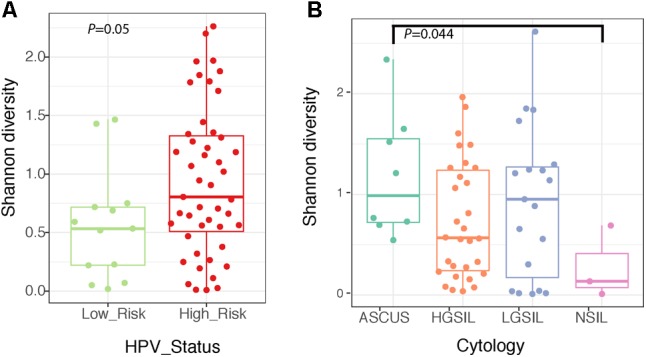
Boxplots depicting Shannon diversity and distinguishing cervical fungal diversity according to HPV status **(A)** and diversity of fungi in the introitus according to cytology **(B)**.

The structure of the fungal community differed significantly according to the identity of the highest-abundance taxon, either Sporidiobolaceae, *Malassezia*, or *Candida* (*P* = 0.001, Figure 6 and Supplementary Table 5). Fungal diversity (Shannon index) was significantly higher in cervical high-risk HPV samples than in those with low risk (Figure 7A; *P* = 0.05). Introitus samples in women with ASCUS showed higher fungal diversity compared to NSIL and HGSIL (*P* = 0.04) (Figure 7B and Supplementary Table 5).

**FIGURE 8:**
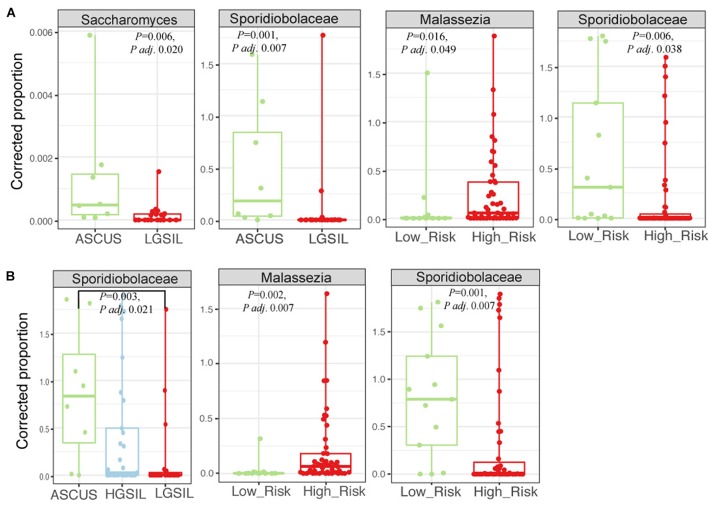
Fungal biomarker signatures in the 58 introitus **(A)** and 55 cervical **(B)** samples. Corrected proportion values correspond to the multiplication of the DNA library concentration with the relative abundance of the OTU- a best practice in fungal microbiota analyses, due to the common fungal taxa that arise from contamination sources.

There were no significant differences in fungal richness with respect to cervical lesions in any of the body sites examined (Supplementary Figures 9A,B and Table 5). *Malassezia* was significantly dominant in introitus samples from high-risk HPV patients (Figure 8A and Supplementary Table 5, *P*adj = 0.049). *Sacharomyces* and Sporidiobolaceae were significantly more dominant in introitus samples from patients with ASCUS compared to LGSIL (Figure 8A, *P*adj = 0.002 and *P*adj = 0.007, respectively). Sporidiobolaceae—the most-abundant fungi present in at least 70% of the samples (Figure 5), was more dominant in cervical samples of patients with ASCUS (*P*adj = 0.021) and low-risk HPV (*P*adj = 0.007) (Figure 8B).

**FIGURE 9:**
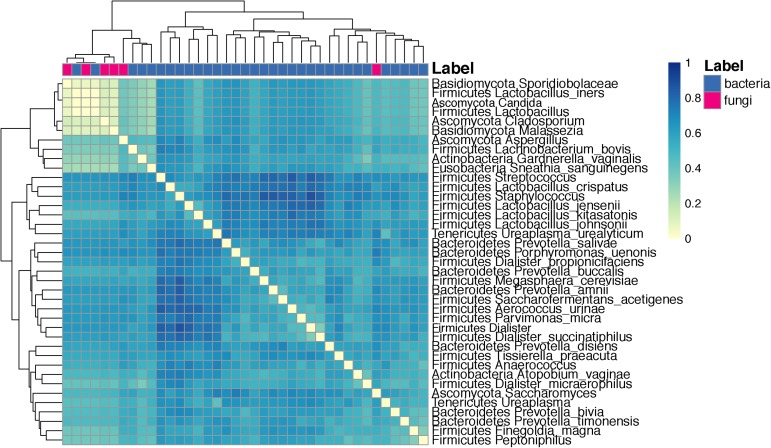
Heat map depicting the analysis of co-occurrence among microbial species and genus level taxa scored using the Dice index. Clustering is done with euclidean distances between each taxa pair using their Dice index across all other taxa.

Co-occurrence analysis using the Dice index indicated that several taxa have a low co-occurrence: Sporidiobolaceae and *L. iners, Candida*, and other *Lactobacillus* species. Conversely, taxa with high co-occurrence included several species of *Lactobacillus* such as *L. crispatus, L. jensenii, L. kitasatonis*, and *L. johnsonii* (Figure 9).

## Discussion

### High Prevalence of HPV and Co-infections in a High-Risk Population

High-risk HPV infections and high-grade cervical lesions have been strongly correlated (Castle et al., 2001). We used a high-resolution approach for HPV detection – SPF_10_ LiPA kit (van Doorn et al., 2002), with similar sensitivities and specificities to other commercial assays (van Hamont et al., 2006; Safaeian et al., 2007), but detects more types of genital HPVs. Differences in HPV prevalence across studies may in part result from the different detection methods used. In Puerto Rico, the prevalence of HPV among urban women aged 18–34 years-old has been reported to be 38.4% in cervical samples and 33.7% in anal physician-collected samples (Ortiz et al., 2013). However, we found in this high-risk urban Hispanic population, a very high HPV prevalence (84%) in cervical lesions (mainly high-risk HPV-types) using the LiPA kit. Other methods maybe be less sensitive, consistent with the lower prevalences reported for Latin America and Caribbean region in high-grade squamous intraepithelial lesions (41–67%) (Parkin et al., 2008). High HPV prevalence ranging from 30 to 50% have been reported for other high-risk Latin American countries, using the same genotyping method SPF_25_/LiPA_10_, including anal specimens in Costa Rica (Castro et al., 2012), and vaginal specimens in Surinam (Geraets et al., 2014), as well as for Nigeria (Adebamowo et al., 2017), Turkey (Barut et al., 2018), and Italy (Carozzi et al., 2018). The very high prevalence found in our study may reflect the high-risk patients from the San Juan Municipality, known to have rising cervical cancer diagnoses (Registro Central De Cáncer De Puerto Rico, 2017). Although we found oncogenic HPV16 to be more abundant in younger and overweight women with HGSIL cervical lesions, these associations were not significant, similar to a prior report that showed HPV prevalence to be comparable between obese and non-obese women (Liu S.H. et al., 2013).

This high-resolution HPV genotyping report for a San Juan population, indicates a high degree of simultaneous low-risk and high-risk infections (with as many as 9 HPV types) associated with diagnostic cervical lesions. Coinfections have been related to the number of sexual partners (Orlando et al., 2012), Co-infections by both low and high risk types have been considered to have antagonistic interactions, possibly reducing the risk of cervical cancer (Luostarinen et al., 1999; Silins et al., 1999). Women co-infected with both high-risk HPV (HPV-16) and low-risk HPV types were shown to have lower risk for invasive squamous cervical cancer and took longer to develop cancer than women infected with hrHPV alone (Sundstrom et al., 2015). Nonetheless, the negative interaction of HPV co-infections has not been shown to apply to other high-risk types.

### Ubiquity and Prevalence of *Lactobacillus iners*

The genital tract of healthy women is known to be dominated by Lactobacilli. This keystone vaginal species produces lactic acid as a fermentation by product and lowers the vaginal pH to ∼3.5–4.5, causing a chemical barrier to pathogens (Boskey et al., 1999). Although we did not use the 16S variable region that typically is used to study *Lactobacillus* (V1–V2) (Gupta et al., 1998; Ravel et al., 2011), we found *L. iners* to be ubiquitous despite its relative abundance varying with HPV risk, cervical lesions or BMI. Changes from normal BMI status are associated with reduced abundance of *L. iners*, which adds to the significant associations of obesity with increased vaginal *Prevotella* diversity (Si et al., 2017). *L. iners* may be more capable of adapting to changing pH and metabolically diverse conditions than other lactobacilli (Macklaim et al., 2011, 2013). The enrichment of cervical *L. kitasatonis* in high-risk HPV type infections and *L. crispatus* in CIN1 and CIN3 lesions also suggests specific niches for these organisms.

### Bacterial Diversity Is Correlated With Advanced CIN Severity but Not With HPV Risk

We did not find significant changes in bacterial richness or diversity with HPV risk. We found an enrichment of *L. kitasatonis* and *T. praeacuta* (not significant) with high-risk HPV. Although we recognize our modest sample size that limits our ability to detect true biomarkers, a recent study with a smaller sample size reported a significant association of non- *L. iners- Lactobacillus* sp. in HPV positive samples, and also no significant differences in richness or diversity in HPV positive samples (Tuominen et al., 2018). Recently, an infection model that mimics immediate early events of the HPV life cycle showed that high risk HPV16 does not provoke many changes in cellular gene expression (Bienkowska-Haba et al., 2018), which may indicate that cellular changes occurring in high and low risk HPV infections may not be sufficient to impact cervicovaginal bacterial communities.

However, we found that bacterial diversity was significantly higher in women with cervical CIN3 lesions, consistent with a prior observation (Mitra et al., 2015). The stromal microenvironment may select for a higher community diversity, possibly due to the marked biochemical and immune differences between CIN lesions and the normal epithelium. Dendritic cell density and phenotypes change with carcinogenesis, coupled with a higher percentage of CD1a+ CD123+ cells and macrophages (Kobayashi et al., 2008). A study of Mexican women suggested *Sneathia* spp. or *Shuttleworthia satelles* (Audirac-Chalifour et al., 2016) as cervical biomarkers for neoplasia, which had previously been related to HPV infections (Lee et al., 2013). Vaginal biomarkers for CIN in Caucasian, Asian and Black women include *Sneathia sanguinegens*, *Anaerococcus tetradius*, and *Peptostreptococcus anaerobius* (Mitra et al., 2015). We also found a positive association of *Prevotella timonensis, P. amnii*, and *P. micra* with CIN3 lesions, taxa previously associated with bacterial vaginosis (Srinivasan et al., 2012). That *P. micra*, an uncommon taxon often found in the mouth, is more prevalent in the cervical lesions, suggests that *P. micra* could seed the cervicovaginal communities via oral sex; this taxon also has also been found in the salivary microbiome of head and neck cancer patients (Guerrero-Preston et al., 2017).

### Fungal Communities Are Significantly Associated With High-Risk HPV and Atypical Squamous Cells of Undetermined Significance

We found dominance of three fungal types: *Candida*, *Malassezia* and Sporidiobolaceae in relation to HPV infection. *Malassezia* are lipophilic cutaneous yeasts, also involved in superficial fungal infections such as pityriasis versicolor (Shams-Ghahfarokhi and Razzaghi-Abyaneh, 2004), atopic dermatitis (Faergemann, 2002) and psoriasis (Paulino et al., 2006). *Malassezia* have been frequently detected in the resident genital microbiota in healthy men, in association with sebaceous (Tyson’s) glands in the lipid-rich prepuce and glans penis (Mayser et al., 2001). *Malassezia* produce bioactive indoles *in vitro* including activators of the aryl hydrocarbon receptor (AhR) (Gaitanis et al., 2008). AhR receptors mediate many skin functions including acceleration of skin development (Loertscher et al., 2002), thus it has been suggested that *Malassezia* may participate in cutaneous carcinogenesis (Gaitanis et al., 2008).

Sporidiobolaceae were significantly associated with low-risk HPV-infections and ASCUS in the introitus and cervix. Sporidiobolaceae are anamorphic yeasts, including *Sporobolomyces*, that has been associated with ulcerative colitis and Crohn’s disease (Ott et al., 2008; Liguori et al., 2016; Qiu et al., 2017). Other abundant fungi enriched in ASCUS included *Sacharomyces* and *Cladosporium*. These associations could reflect gut contamination arising from sexual practices, which may relate to vaginal microbiome changes (Noyes et al., 2018).

Fungi can colonize perturbed niches, as seen after the introduction of *C. albicans* to mice after antibiotic treatment caused significant shifts to the gut microbiota (Erb Downward et al., 2013). Conversely, depletion of commensal intestinal fungi with anti-fungals may trigger growth of pathogenic bacterial microbiota (Qiu et al., 2015). *C. albicans* are the most prevalent species in vulvovaginal candidiasis, with enhanced proteolytic activity and antigen modulation to penetrate the mucosal surface (Ganguly and Mitchell, 2011). Although correlating the fungal and bacterial dynamics in the human body has received little attention, we showed here that *Lactobacillus*, *Malassezia*, and *Candida* have a low co-ocurrence. This suggests that either the reduction in *Lactobacillus* may result in an opportunity for fungi to colonize the cervicovaginal niche or, alternatively, these specific fungal infections create an environment that inhibits Lactobacilli growth. Our work, although on a small scale, illustrates the complex relationship between the mycobiome and the bacterial microbiota in the vagina and cervix during HPV infections.

## Conclusion

To our knowledge, no other studies simultaneously assess HPV genotype diversity, bacterial and fungal communities associated with different histopathologic features. Despite the modest sample size, our data suggests that the structure of the cervicovaginal bacterial biota is correlated with CIN while the mycobiota is correlated with both high-risk HPV infections and CIN severity, in this reproductive-age Hispanic population. Characterizing the structural features of the cervicovaginal ecosystem may be an important step in integrating understanding of the biology of cervical neoplasia, to better develop therapeutic interventions that target the microbiota.

## Author Contributions

FG-V conceived and designed the experiments, performed the experiments, analyzed the data, contributed reagents, materials, analysis tools, wrote the paper, prepared figures and/or tables, and was the responsible for funding. JR recruited the patients, acquired the samples and patient metadata and reviewed drafts of the paper. CZ helped analyze the data and reviewed drafts of the paper. DV-R and GO-M helped analyze data and reviewed drafts of the paper. FV-S helped in the patient recruitment process and reviewed drafts of the paper. MS-V and MM-F genotyped HPV and reviewed drafts of the paper. MG-C helped in the patient recruitment process and metadata acquisition, reviewed drafts of the paper. JW was responsible for Resphera Insight taxonomic assignments and reviewed drafts of the paper. KB, MD-B, and MB provided insights to the analyses, reviewed thoroughly drafts of the paper.

## Conflict of Interest Statement

The authors declare that the research was conducted in the absence of any commercial or financial relationships that could be construed as a potential conflict of interest.

## Footnotes

1 http://www.earthmicrobiome.org/emp-standard-protocols/16s/

2 https://github.com/kylebittinger/brocc
