# Supplementary material for: Cervicovaginal Fungi and Bacteria Associated With Cervical Intraepithelial Neoplasia and High-Risk Human Papillomavirus Infections in a Hispanic Population
Source: Front Microbiol. 2018 Oct 23;9:2533. doi: 10.3389/fmicb.2018.02533 (PMC6208322; doi:10.3389/fmicb.2018.02533)
Supplement: Supplementary file 3 [file Table_3.DOCX]

**Supplementary Table 3. Number of 16S and ITS reads according to HPV status for the 340 samples (170 for each 16S and ITS dataset) of the 58 patients.**

| **Sampling_Site** | **HPV_Status^1^** | **Patient samples^2^** | **Total # of 16S Reads** | **Total # of 16S OTUs** | **Total # of ITS reads** | **Total # of ITS OTUs** |
| --- | --- | --- | --- | --- | --- | --- |
| **Introitus** | High_Risk | 13 | 479,162 | 965 | 377,199 | 160 |
|  | Low_Risk | 45 | 1,306,631 | 2,845 | 1,066,075 | 257 |
| **Cervix** | High_Risk | 12 | 619,719 | 843 | 385,199 | 156 |
|  | Low_Risk | 43 | 1,264,775 | 3,080 | 895,781 | 256 |
| **Anus** | High_Risk | 12 | 789,270 | 9,562 | 372,393 | 156 |
|  | Low_Risk | 45 | 3,953,433 | 14,984 | 1,262,051 | 257 |

**^1^** HPV negative and HPV low-risk were grouped together in the “Low Risk” group. Low-Risk HPVs were defined as detected genotypes 6, 34, 42, 43, 44, 53 and 74, while the High-risk detected types were 16, 18, 31, 33, 39, 45, 51, 52, 56, 66, 68/73.

**^2^** Only bacterial and fungal samples which had >1,000 reads were used in the analyses: introitus 58 samples, cervix 55 and anus 57.
