# Supplementary material for: Cervicovaginal Fungi and Bacteria Associated With Cervical Intraepithelial Neoplasia and High-Risk Human Papillomavirus Infections in a Hispanic Population
Source: Front Microbiol. 2018 Oct 23;9:2533. doi: 10.3389/fmicb.2018.02533 (PMC6208322; doi:10.3389/fmicb.2018.02533)
Supplement: Supplementary file 4 [file Table_4.DOCX]

| **HPV-Risk** | **Cervical Biopsy*** | **Introitus Samples** | **Introitus bacterial reads** | **Introitus bacterial OTUs** | **Introitus fungal reads** | **Introitus fungal OTUs** | **Cervical Samples** | **Cervical bacterial reads** | **Cervical bacterial OTUs** | **Cervical fungal reads** | **Cervical fungal OTUs** |
| --- | --- | --- | --- | --- | --- | --- | --- | --- | --- | --- | --- |
| High- Risk | No Lesion | 4 | 166,035 | 853 | 159,908 | 66 | 5 | 149,834 | 1,662 | 120,682 | 70 |
|  | CIN 1 | 10 | 383,763 | 1,035 | 196,026 | 140 | 9 | 406,184 | 739 | 158,770 | 131 |
|  | CIN3 | 23 | 659,635 | 1,663 | 523,857 | 216 | 21 | 644,459 | 1,589 | 452,344 | 203 |
|  | Not recorded | 8 | 115,972 | 966 | 186,284 | 122 | 8 | 64,298 | 442 | 163,985 | 122 |
| Low- Risk | No Lesion | 5 | 173,318 | 362 | 164,625 | 70 | 5 | 319,091 | 406 | 176,366 | 70 |
|  | CIN 1 | 3 | 140,552 | 461 | 41,151 | 56 | 1 | 120,110 | 295 | 67,601 | 54 |
|  | CIN3 | 4 | 152,768 | 480 | 150,773 | 66 | 4 | 175,678 | 389 | 124,166 | 66 |
|  | Not recorded | 4 | 166,035 | 853 | 159,908 | 66 | 5 | 149,834 | 1662 | 120,682 | 70 |

**Supplementary Table 4. Number of reads according to HPV status and biopsy lesion used for the cervical and introitus microbiota analyses**
