# Supplementary material for: Cervicovaginal Fungi and Bacteria Associated With Cervical Intraepithelial Neoplasia and High-Risk Human Papillomavirus Infections in a Hispanic Population
Source: Front Microbiol. 2018 Oct 23;9:2533. doi: 10.3389/fmicb.2018.02533 (PMC6208322; doi:10.3389/fmicb.2018.02533)
Supplement: Supplementary file 6 [file Data_Sheet_1.PDF]

Cervicovaginal fungi and bacteria associated with cervical intraepithelial neoplasia and high-risk Human Papillomavirus infections in a Hispanic population

Supplementary Figures

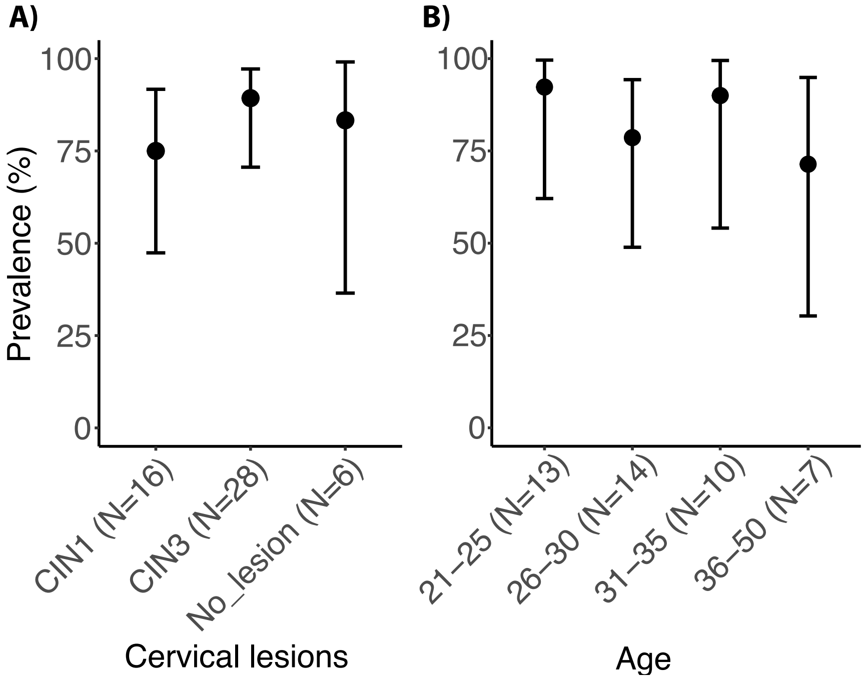

Supplementary Figure 1. HPV prevalence by positive and negative cervical lesions (A) and by age categories, only among lesion-positive women (B). No significant differences were observed for both analyses (Fisher´s Exact Test, p>0.05).

A)

Cytology	ASCUS	HGSIL	LGSIL	NSIL
N women	6	25	18	3
HPV16_H	0.00	0.40	0.22	0.00
HPV18_H	0.17	0.16	0.00	0.00
HPV31_H	0.00	0.16	0.17	0.33
HPV33_H	0.17	0.16	0.00	0.00
HPV39_H	0.00	0.00	0.06	0.00
HPV45_H	0.17	0.04	0.00	0.00
HPV51_H	0.00	0.12	0.11	0.00
HPV52_H	0.17	0.08	0.00	0.33
HPV56_H	0.00	0.04	0.00	0.00
HPV6	0.00	0.12	0.11	0.33
HPV11	0.00	0.04	0.06	0.00
HPV34	0.17	0.04	0.28	0.33
HPV35	0.17	0.12	0.22	0.33
HPV42	0.17	0.16	0.28	0.00
HPV43	0.00	0.04	0.00	0.33
HPV44	0.00	0.08	0.17	0.33
HPV53	0.00	0.20	0.28	0.33
HPV54	0.00	0.12	0.06	0.00
HPV66	0.17	0.16	0.22	0.33
HPV68	0.00	0.00	0.00	0.00
HPV63_74	0.00	0.00	0.00	0.00

B)

BMI_Status	Underweight	Normal	Overweight	Obese
N women	3	19	18	12
HPV16_H	0.33	0.26	0.33	0.17
HPV18_H	0.00	0.16	0.11	0.00
HPV31_H	0.00	0.11	0.22	0.17
HPV33_H	0.33	0.05	0.11	0.08
HPV39_H	0.00	0.11	0.11	0.08
HPV45_H	0.00	0.11	0.00	0.00
HPV51_H	0.00	0.05	0.17	0.33
HPV52_H	0.00	0.05	0.22	0.33
HPV56_H	0.67	0.05	0.11	0.08
HPV6	0.00	0.11	0.22	0.17
HPV11	0.00	0.05	0.06	0.08
HPV34	0.00	0.00	0.06	0.00
HPV35	0.00	0.11	0.00	0.00
HPV42	0.33	0.00	0.06	0.17
HPV43	0.00	0.05	0.00	0.00
HPV44	0.00	0.05	0.17	0.17
HPV53	0.00	0.26	0.17	0.17
HPV54	0.33	0.00	0.00	0.08
HPV66	0.00	0.26	0.22	0.17
HPV68	0.00	0.21	0.00	0.00
HPV63_74	0.33	0.11	0.33	0.08

C)

Cervical lesior	CIN1	CIN3	No_lesion
N women	12	24	5
HPV16_H	0.17	0.42	0.40
HPV18_H	0.08	0.17	0.00
HPV31_H	0.17	0.25	0.00
HPV33_H	0.00	0.17	0.00
HPV39_H	0.08	0.08	0.20
HPV45_H	0.08	0.04	0.00
HPV51_H	0.33	0.04	0.40
HPV52_H	0.17	0.17	0.20
HPV56_H	0.08	0.13	0.20
HPV6	0.08	0.21	0.40
HPV11	0.00	0.08	0.00
HPV34	0.00	0.00	0.20
HPV35	0.08	0.00	0.00
HPV42	0.00	0.13	0.00
HPV43	0.00	0.00	0.00
HPV44	0.08	0.13	0.20
HPV53	0.25	0.17	0.20
HPV54	0.00	0.04	0.00
HPV66	0.25	0.25	0.20
HPV68	0.08	0.08	0.00
HPV63_74	0.17	0.21	0.40

D)

Age_Range	21-25	26-30	31-35	36-50
N women	14	12	19	7
HPV16_H	0.43	0.33	0.16	0.14
HPV18_H	0.00	0.00	0.21	0.14
HPV31_H	0.14	0.25	0.11	0.00
HPV33_H	0.07	0.17	0.11	0.00
HPV39_H	0.14	0.00	0.11	0.14
HPV45_H	0.07	0.00	0.05	0.00
HPV51_H	0.21	0.08	0.11	0.14
HPV52_H	0.21	0.17	0.11	0.14
HPV56_H	0.21	0.08	0.00	0.14
HPV6	0.14	0.25	0.11	0.14
HPV11	0.00	0.08	0.11	0.00
HPV34	0.07	0.00	0.00	0.00
HPV35	0.00	0.00	0.05	0.14
HPV42	0.00	0.08	0.11	0.00
HPV43	0.00	0.00	0.00	0.14
HPV44	0.14	0.08	0.11	0.00
HPV53	0.21	0.17	0.21	0.14
HPV54	0.00	0.08	0.00	0.00
HPV66	0.21	0.17	0.21	0.14
HPV68	0.14	0.00	0.05	0.14
HPV63_74	0.21	0.08	0.16	0.29

Supplementary Figure 2. Heat maps of HPV relative abundances according to A) Cytology, B) BMI status, C) Biopsy and D) Subject age in 52 women. There are no significant differences of HPV genotype abundances for any category ($p>0.05$, Fisher's Exact Test).

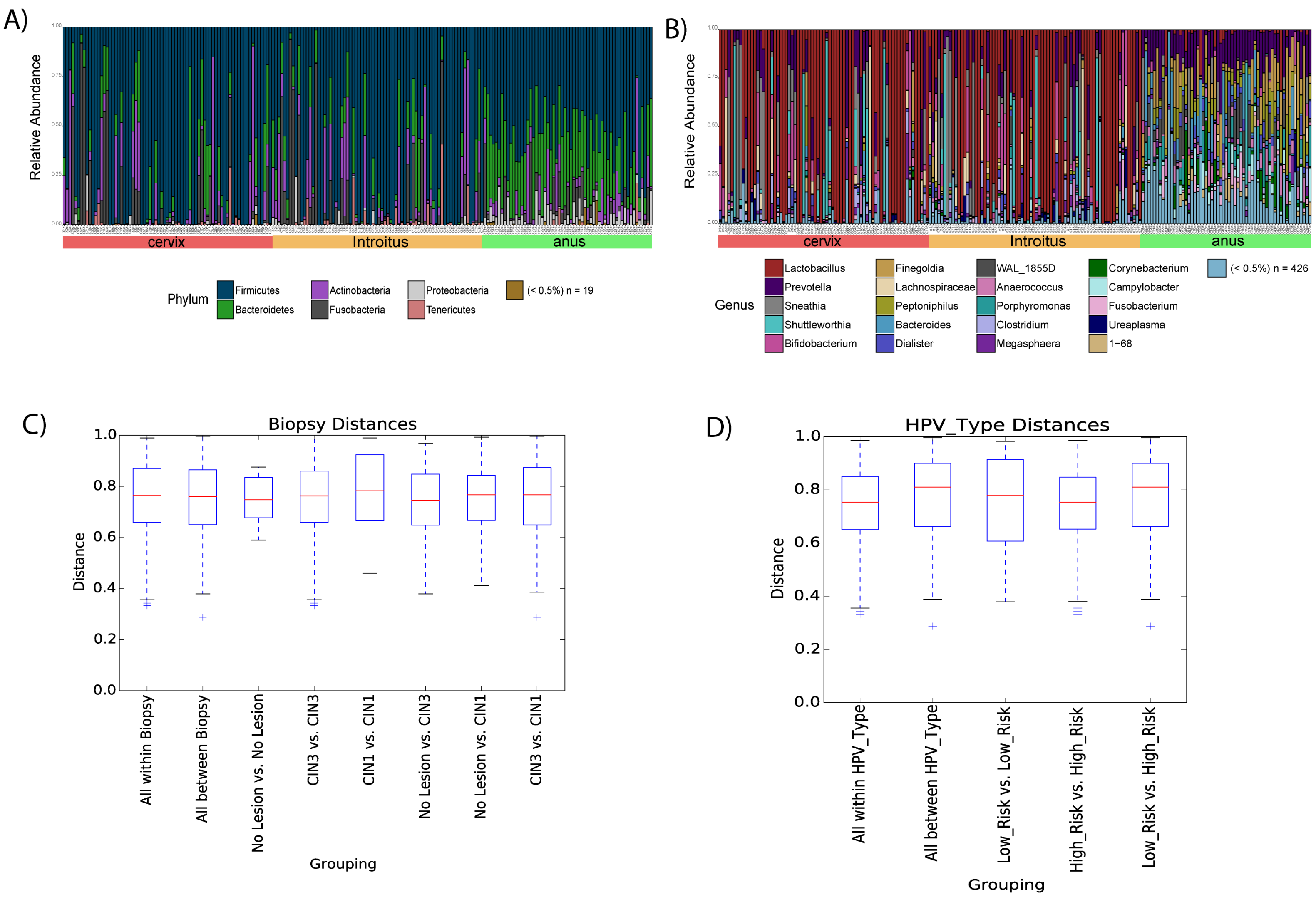

Supplementary Figure 3. Taxonomic plots of all body sites at the phylum and genus levels (A, B) and UniFrac distance plots among anal samples according to Biopsy and HPV type distances

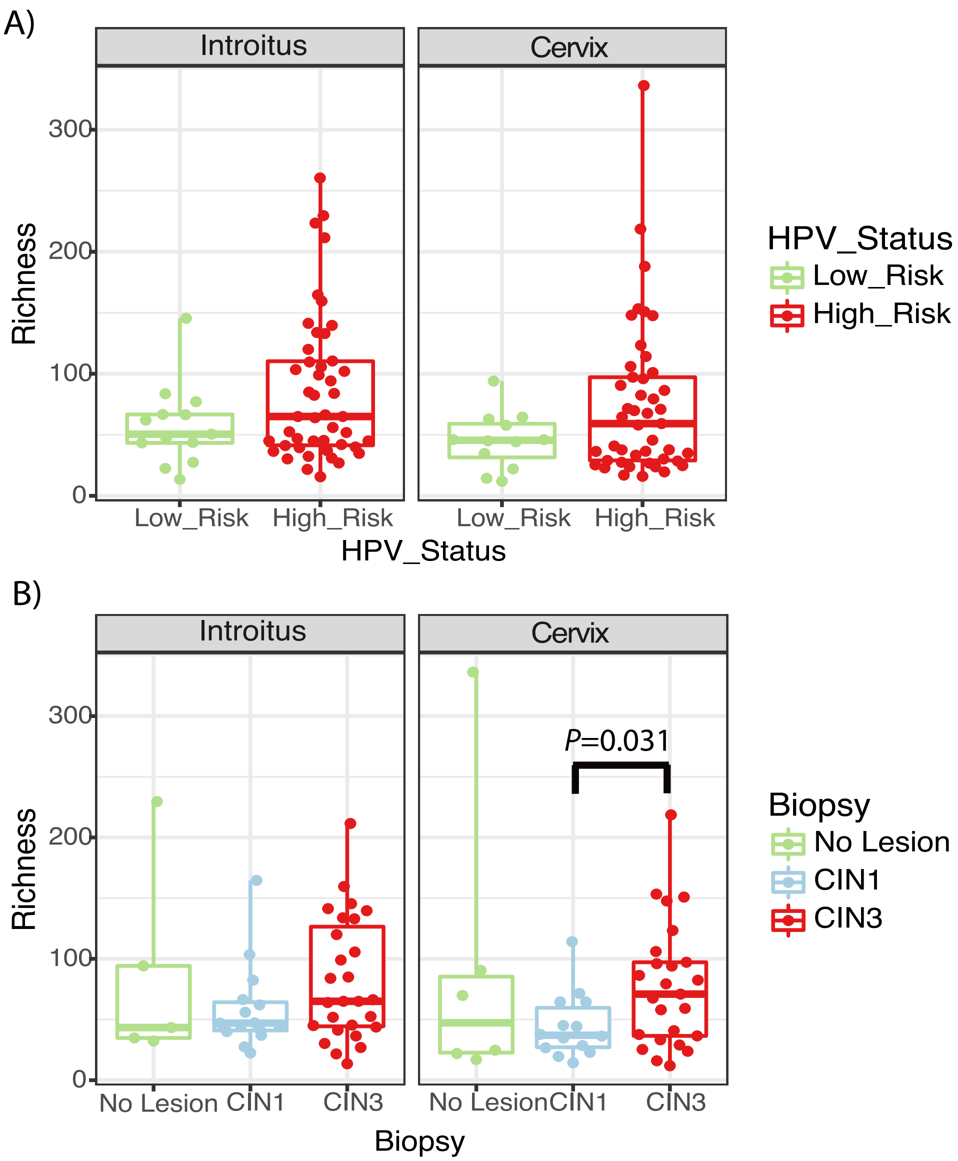

Supplementary Figure 4. Boxplots of observed OTU richness for Introitus and Cervix based on HPV status (A) and Biopsy findings (B). Analyses was done at a rarefaction level of 5,000 sequences. Cervical bacterial richness was higher for CIN3 versus CIN1 patient samples (P= 0.031, see Supplementary Table 5).

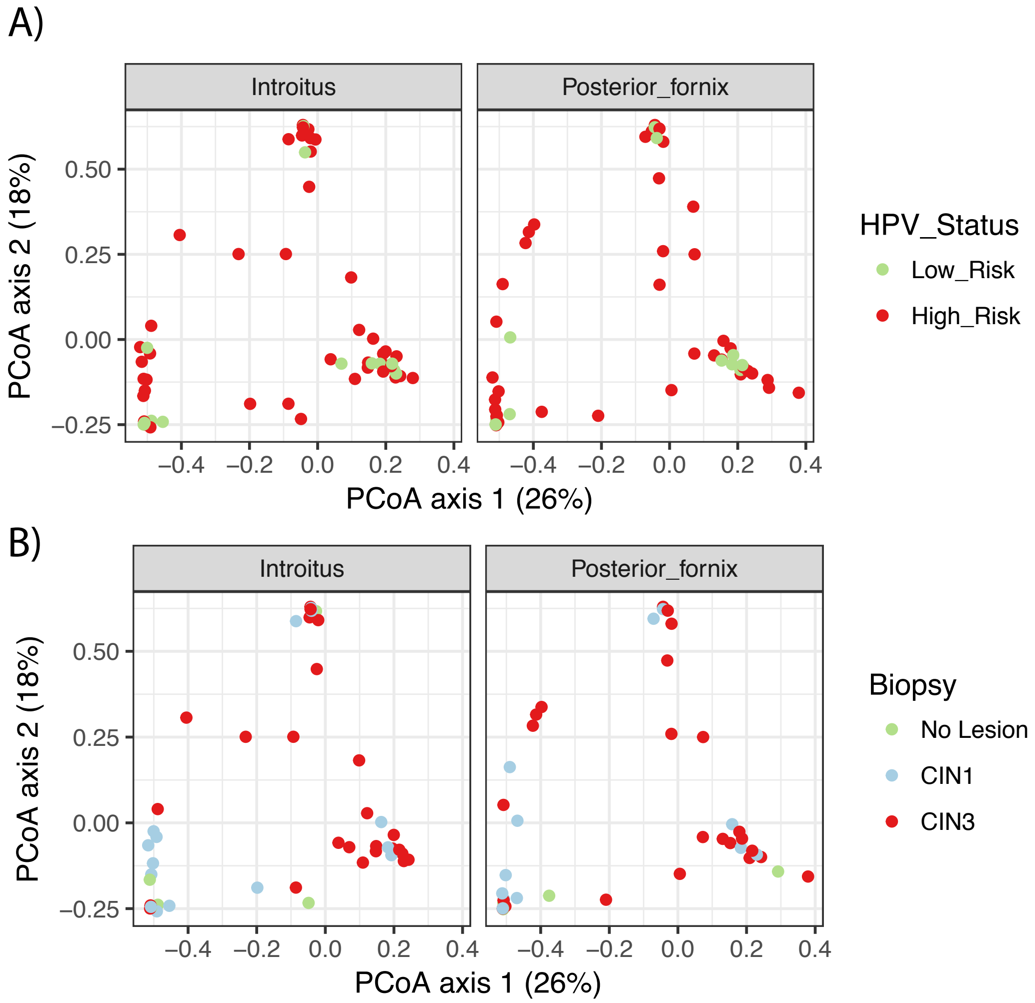

Supplementary Figure 5. Beta diversity analyses of the 58 introitus and 55 cervical 16S patient samples according to HPV status and Biopsy status. PCoA was built using Bray-Curtis distances. Statistical tests for differences in group centroid position did not reveal any significant differences between HPV status ($P>0.05$) although there were significant differences between samples without lesions and CIN3 (($P=0.006$) (see Supplementary Table 5).

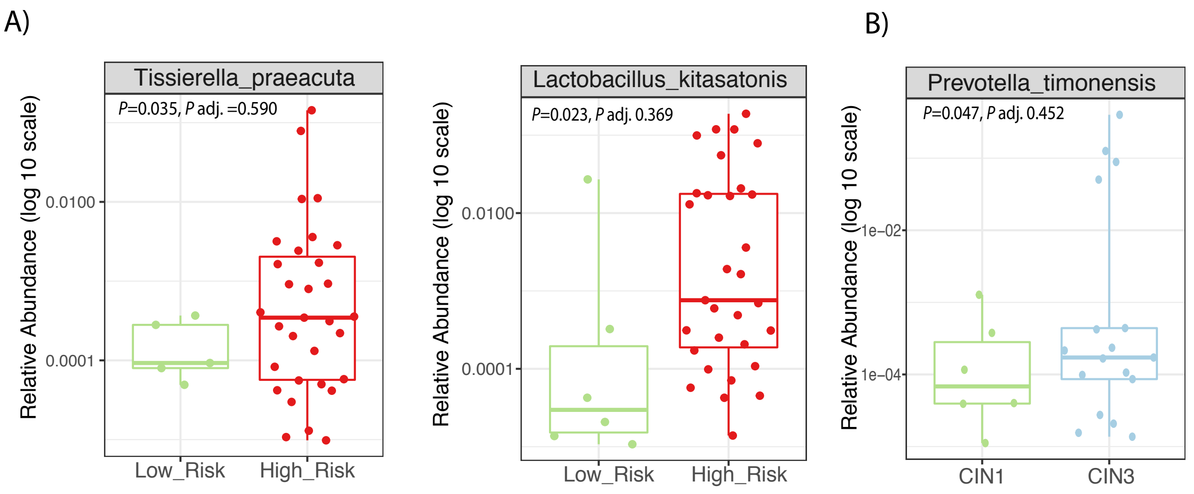

Supplementary Figure 6. Selected bacterial signatures enriched in HPV-risk (A) and biopsy categories (B) in the introitus samples (Tissierella praeacuta) and in Cervical samples - *Lactobacillus kitasatonis* and *P. timonensis*.

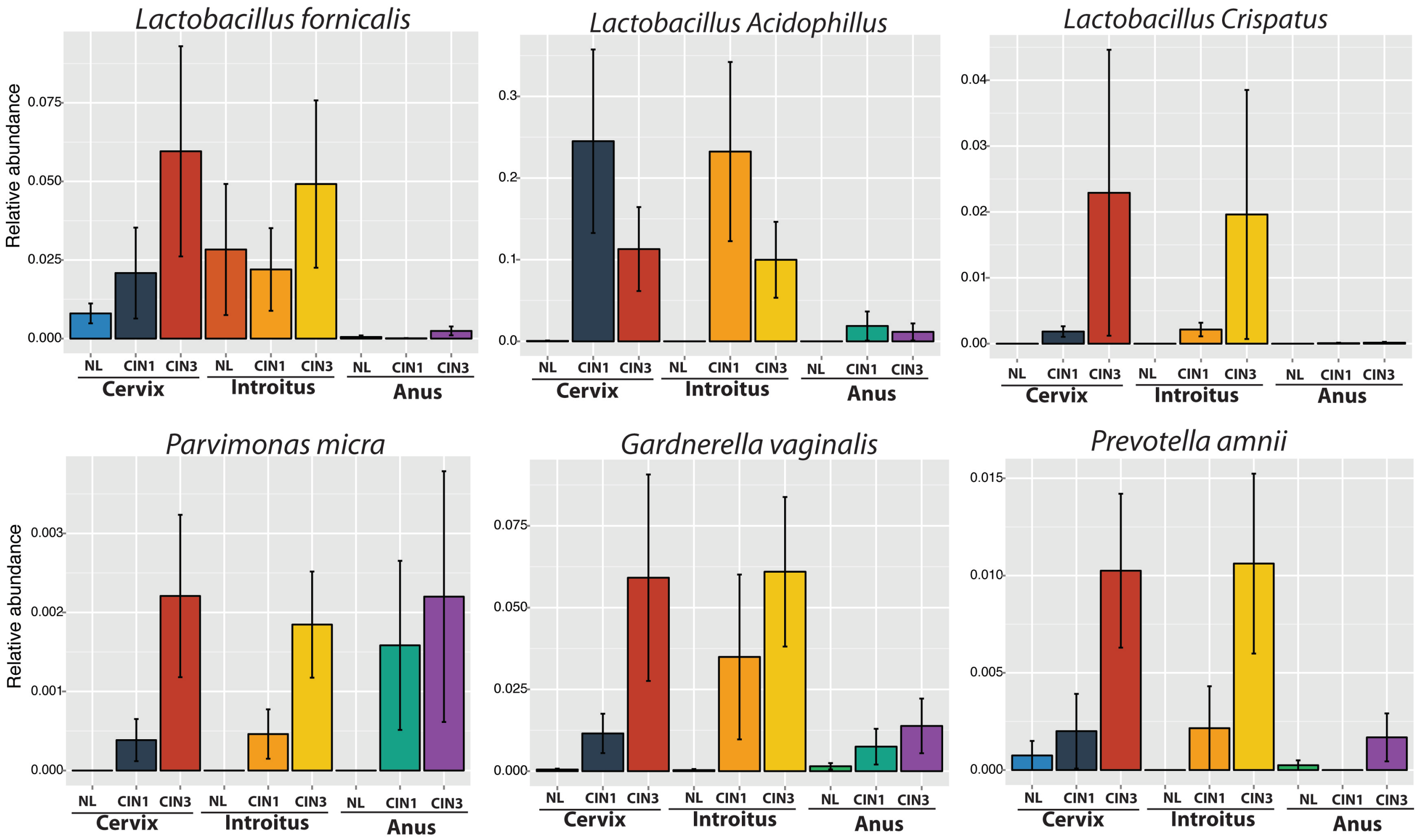

Supplementary Figure 7. Box-plots representing selected taxa that show an increase from no-lesion to CIN1 and CIN3 in most body sites (none of these changes are significant).

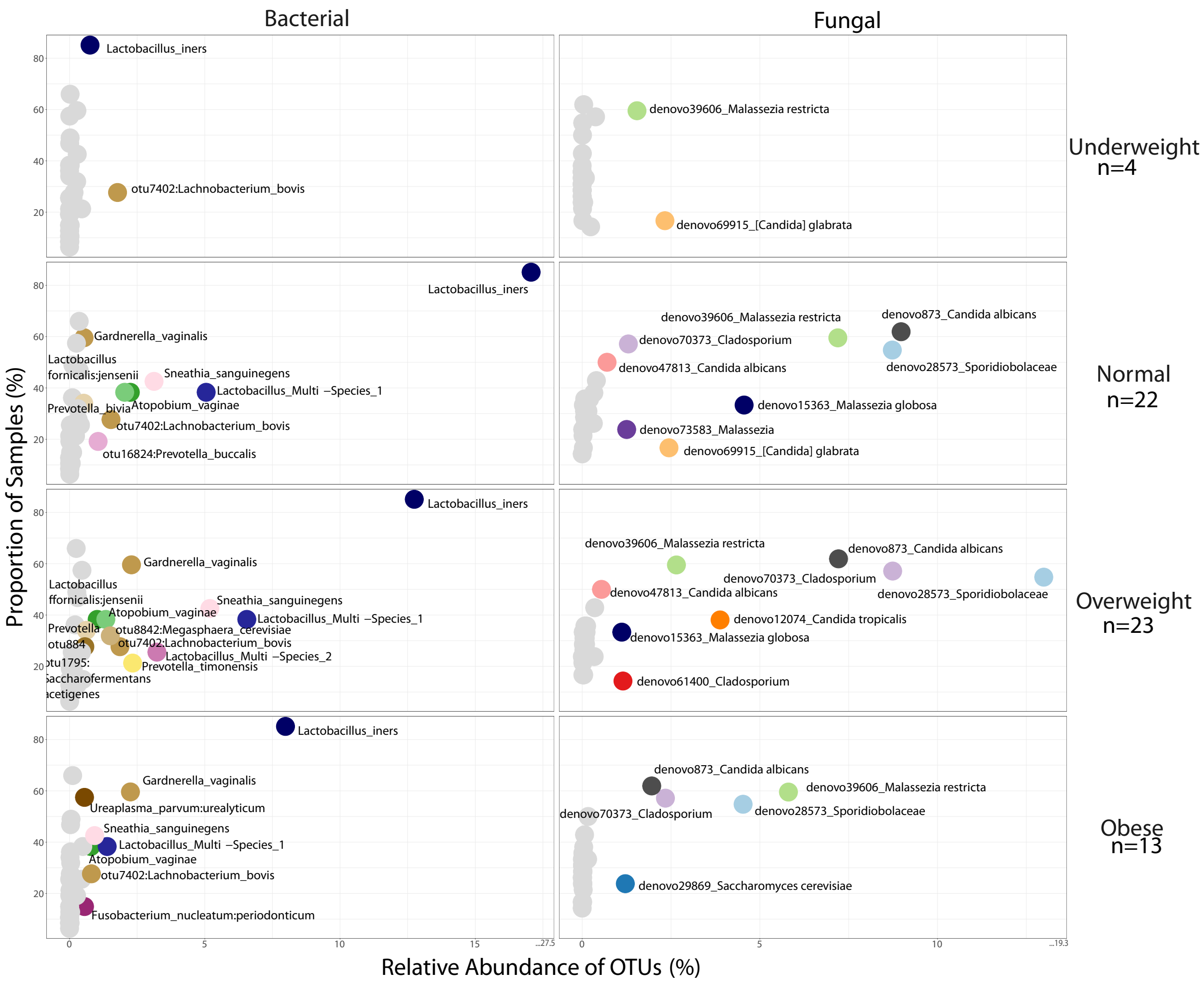

Supplementary Figure 8. Ubiquity dot plot representing bacterial and fungal OTUs in the introitus, distributed according to their relative abundance and ubiquity, for the different BMI categories. The analyses were based on the transformation of the OTU table with the *melt* function into single columns for relative abundance, ubiquity and domain using package *reshape2*. The dot plot was built using the *ggplot2* package using the facet approach to partitioning the plot into multiple panels according to the metadata variables.

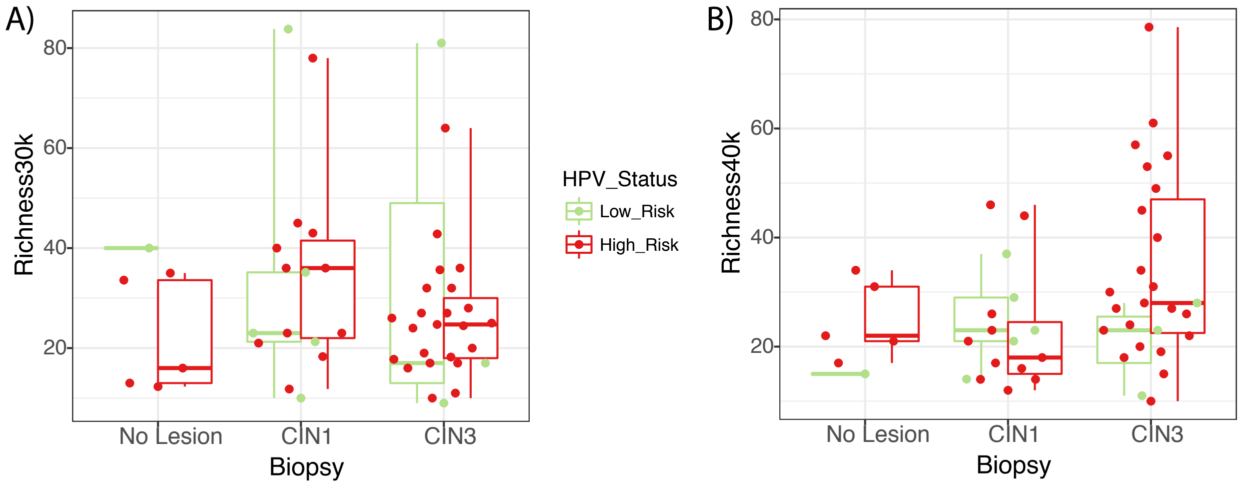

Supplementary Figure 9. Fungal richness in Introitus (A) and Cervix (B) samples based on HPV status and biopsy status. Analyses were done at rarefaction levels of 30,000 sequences for introitus and 40,000 sequences for cervix samples, and shown as median $\pm$ IQR $\pm$ 95% CI. Richness was not significantly higher for any of the comparisons (p-value > 0.05).
